# Training Based on Electrical Stimulation Superimposed Onto Voluntary Contraction Would be Relevant Only as Part of Submaximal Contractions in Healthy Subjects

**DOI:** 10.3389/fphys.2018.01428

**Published:** 2018-10-12

**Authors:** Thierry Paillard

**Affiliations:** Laboratoire Mouvement, Equilibre, Performance et Santé (UPRES EA 4445), University of Pau and Pays de l'Adour, Pau, France

**Keywords:** muscle, electrical stimulation, training, voluntary contraction, superimposed stimulation, submaximal intensity, exercise, performance

Voluntary (VOL) and electrically stimulated (ES) muscular contractions engender differences in activation of muscle fibers and metabolism (Vanderthommen and Duchateau, 2007). During submaximal VOL actions, even if the muscle fibers activated are distributed in the whole muscle, they are progressively recruited in an orderly fashion from small to large according to the intensity of the contraction considered (Henneman et al., 1965), i.e., from the viewpoint of distribution of muscle fibers in quadriceps femoris mainly from the depth of the muscle to the surface since small fibers (slow-twitch fibers, tonic fibers, or type I fibers) are mainly located in the depth of the muscle while large fibers (fast-twitch, phasic fibers, or type II fibers) are mainly located in the surface (Lexell et al., 1983). In turn, the muscle fibers recruitment through ES depends on the current density and it mainly involves muscle fibers located directly beneath the stimulation electrodes since the current density decreases with increasing depth of muscle. Muscle fibers are recruited from the surface of the muscle to the depth according to the current intensity. The higher the intensity, the deeper the fibers are recruited independently of the type of fibers (and the excitability threshold linked to their size) which means that the muscle fibers recruitment is random and spatially fixed (Feiereisen et al., 1997; Vanderthommen et al., 2003; Gregory and Bickel, 2005). Moreover, ES can enhance energy consumption, carbohydrate oxidation, and whole body glucose uptake at low intensity of exercise substantially more than VOL (Hamada et al., 2004). Overall, VOL and ES can be considered as complementary stimuli of a different nature, inducing different acute physiological effects.

Theoretically, the simultaneous superimposition of ES onto VOL (VOL + ES) should augment the produced force through additional muscle fibers recruitment in acute application, and should constitute a potential accumulation (possible additional gains) of the physiological effects induced by each contraction in terms of improvement of muscular power, strength or endurance in the context of chronic application.

Practically, acute application of VOL + ES in pathological (e.g., injured) or over-trained (e.g., chronically fatigued) subjects presenting incomplete voluntary (central) activation levels (i.e., unable to fully activate their muscle) indeed facilitates additional muscle fibers recruitment or muscle fibers firing rates and thus enables an increase in production force in comparison with VOL (Koutedakis et al., 1995). In return, with healthy subjects who are able to fully activate their muscles, VOL + ES does not generate any enhancement of the force production in comparison with VOL (e.g., Hortobägyi et al., 1992). Chronic application of VOL + ES, with pathological subjects following post-traumatic rehabilitation programs (e.g., related to arthroplasty, arthroscopy, ligamentoplasty), is more effective than VOL to facilitate recovery of injuries (e.g., Drapper and Ballard, 1991). VOL + ES compensates for volume and muscle strength deficit with more efficiency than programs using VOL or ES separately (Paillard et al., 2005). With healthy subjects, VOL + ES does not reveal significant benefits in comparison with programs performed only with VOL or ES (e.g., Paillard et al., 2004). In fact, most of muscle fibers are already activated with VOL and superimposed electrical stimulation does not enable the supplementary recruitment of muscle fibers and cannot induce greater long-term training adaptations (Wirtz et al., 2015). Whether in acute or chronic application, VOL + ES does not result in any advantage in comparison with VOL or ES when subjects are healthy and their central nervous system (CNS) fully activates their skeletal muscles and their locomotor apparatus is devoid of any pathology (Paillard et al., 2005).

Since the publication of the review article by Paillard et al. (2005), the understanding of VOL + ES as a training technique has only slightly evolved. Indeed, there is still a certain consensus according to which VOL + ES would be not more efficient than VOL (with or without additional weight/load) or ES alone in order to improve motor and/or sport performance in chronic application whether with isometric, dynamic or plyometric movements (Paillard et al., 2005; Herrero et al., 2010; Park et al., 2016; Wirtz et al., 2016; Gomes da Silva et al., 2018). Yet, some recent papers showed that VOL + ES could bring some advantages in comparison with VOL and ES practiced alone as part of training programs aiming at improving motor performance in healthy subjects (Wahl et al., 2012, 2014, 2015; Matsuse et al., 2013; Mathes et al., 2017). Hence, it seems relevant to analyze why some studies reported benefit effects of VOL + ES in chronic application in comparison with VOL and ES in healthy subjects.

In fact, for regularly repeated maximal tasks (maximal resistance/strength exercises), it was confirmed that VOL + ES does not enable the force produced to be increased after a training period (e.g., Park et al., 2016). In turn, as part of regularly repeated submaximal tasks (submaximal resistance/strength exercises), VOL + ES could improve motor performance more than VOL or ES alone after a training period. Evidence suggests that submaximal tasks engender greater muscle fibers recruitment with VOL + ES than with VOL or ES (Figure 1) and would be likely to generate greater gains in terms of motor output after a training period.

**Figure 1:**
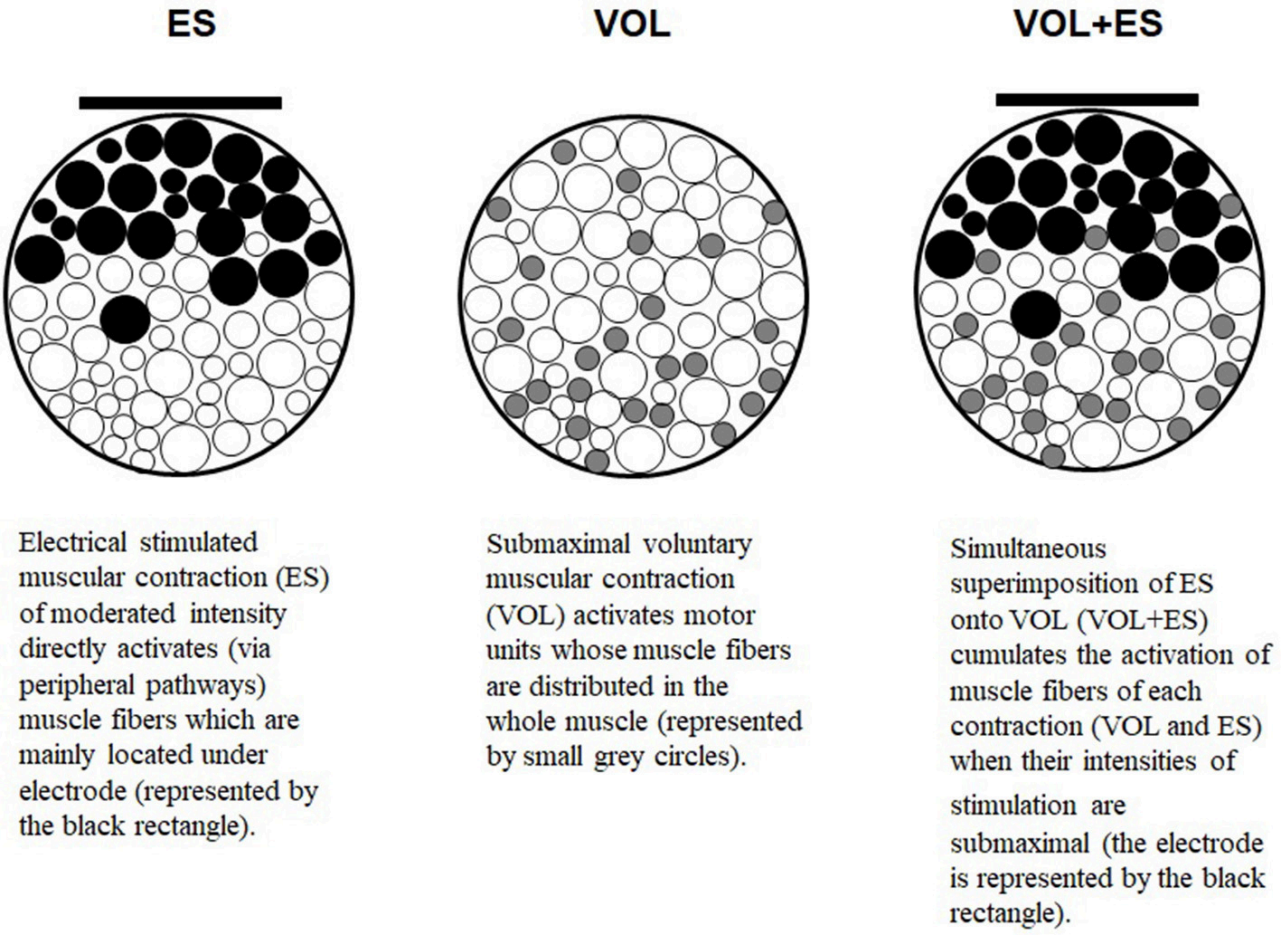
When VOL and ES are applied in submaximal condition (i.e., submaximal intensity), the superimposition of ES onto VOL enables a greater muscle fibers recruitment than the completion of VOL or ES alone.

To this end, as part of the VOL + ES application, on the one hand, the voluntary contraction should be relatively remote from maximal effort (submaximal intensity) and on the other hand, the intensity of the current related to electrical stimulation should be relatively low (e.g., 15–25 mA <) in order to allow any well-coordinated movements. Only submaximal contractions enable an efficient movement control with VOL + ES (Bezerra et al., 2011). If the intensity of the current applied to motor muscles is too high, no free and accurate segmental displacement of limbs is achievable since the resistance exerted on joints is too strong, which impedes or limits movement. The intensity value requires to render possible motor/sport activity of subject while being electrically stimulated. Moreover, the VOL + ES application in submaximal condition (i.e., submaximal contraction) would induce greater metabolic activation and energy consumption as well as greater muscle fibers recruitment and motor output during exercise in comparison with the VOL application. All these different physiological changes would not occur in maximal motor tasks i.e., maximal contractions (Paillard et al., 2005).

From a metabolic viewpoint, some studies showed that acute metabolic changes (e.g., some respiratory, cardiac, biological and biochemical blood parameters) induced by exercise as well as energetic and mechanical output are greater during cycling with superimposed ES than during cycling alone (Wahl et al., 2012, 2014, 2015; Matsuse et al., 2013; Mathes et al., 2017). Hence, one can infer that ES during cycling exercise might be an enhancing stimulus for skeletal muscle metabolism and induced adaptations. Wahl et al. (2014) concluded that at low exercise intensities, VOL + ES characterizes a high stimulus by provoking greater hormonal secretions (e.g., cortisol, Growth Hormone). This high stimulus would entail adaptations related to metabolic endurance (e.g., expression of aerobic enzymes via cortisol, erythropoiesis via Growth Hormone). These favorable enzymatic and hormonal responses were not observed at high intensities in comparison with VOL (Wirtz et al., 2015). Moreover, VOL + ES at submaximal intensity would enhance glucose metabolism through additional fast-twitch muscle fibers recruitment in comparison with VOL (Watanabe et al., 2014). Overall, long-term training adaptations induced by VOL + ES at submaximal intensity contribute to positive effects, similar to those of VOL intense trainings (Wahl et al., 2014) provided that the training period is sufficiently long e.g., more than 4 weeks (Mathes et al., 2017).

From a motor output viewpoint, other authors reported that at submaximal intensity, VOL + ES would be also more efficient than VOL (Valli et al., 2002). They indeed showed that the superimposition of ES onto submaximal contractions (60% of maximal voluntary contraction) induced better strength gains than the same exercise performed without ES superimposition. Theses authors hypothesized that the superimposition of ES with submaximal contractions induced a neurogenic facilitatory effect enabling greater strength development thanks to the recruitment of supplementary muscle fibers. This type of training would be relevant not only for the ipsilateral limb but also for the contralateral limb. Indeed, Bezerra et al. (2009) observed that VOL + ES would cause additional training effects and greater cross-education compared with VOL training, because it would activate the same neural pathways that are used normally in voluntary exercise, with additional afferent inputs (centrally integrated) provoked by the electrostimulation. The review article by Frazer et al. (2018) linked to neural adaptations as part of the cross-education would reinforce this assumption.

Moreover, the quality of a training program depends on its intensity (knowing that the quantity of a training program depends on its amount). It is well-known that the intensity is fundamental in order to improve motor performance. However, it is not always possible to constantly train sportsmen at high intensity because they would risk overtraining and chronic fatigue (Lehmann et al., 1992; Anish, 2005; Purvis et al., 2010). Hence, the intensity should be regularly reduced to avoid the harmful consequences of excess stimulation of the CNS (Kellmann, 2010; Schaun et al., 2018). Based on this data, in order to apply a certain intensity, regularly or occasionally, by limiting the involvement of the CNS (i.e., central factors) while maintaining a strong stimulation of motor muscles (i.e., peripheral factors), the superimposition of ES onto VOL can be used as part of training aiming the improvement/maintaining of muscle strength or endurance. Indeed, even if the ES exercise affects the CNS (i.e., corticospinal excitability) in acute application (Chaubet et al., 2013; Kotan et al., 2015) a VOL + ES fatiguing exercise impaired motor output (e.g., muscle strength) and motor control (e.g., postural control) less than did a VOL fatiguing exercise (Paillard et al., 2010). These authors suggested that the contribution of VOL + ES would limit the changes in muscle activation and then the central fatigue during a fatiguing exercise performed with submaximal contraction. In practice, besides its beneficial effects generating greater physiological adaptations compared to VOL, VOL + ES may limit muscle fatigue in acute application which may reduce the risk of overtraining in chronic application.

VOL + ES would present few if any advantage as part of motor/sport performance when it is applied at maximal intensity. In return, at submaximal intensity, VOL + ES could constitute an interesting and complementary training technique to traditional training in terms of improvement of motor/sport performance as well as reduction of residual fatigue. Other works should be achieved in order to confirm or invalidate these hypotheses.

## Author contributions

The author confirms being the sole contributor of this work and has approved it for publication.

### Conflict of interest statement

The author declares that the research was conducted in the absence of any commercial or financial relationships that could be construed as a potential conflict of interest.
